# Acknowledgment to Reviewers of *Metabolites* in 2020

**DOI:** 10.3390/metabo11020068

**Published:** 2021-01-26

**Authors:** Metabolites Editorial Office

**Affiliations:** MDPI AG, St. Alban-Anlage 66, 4052 Basel, Switzerland

Peer review is the driving force of journal development, and reviewers are gatekeepers who ensure that *Metabolites* maintains its standards for the high quality of its published papers. Thanks to the cooperation of our reviewers, in 2020, the median time to first decision was 15 days and the median time to publication was 40 days. The editors would like to express their sincere gratitude to the following reviewers for their precious time and dedication, regardless of whether the papers were finally published:
Abbriano, RaffaelaLiu, RunxiaAbdallah, MohamedLiu, YouzhongAbu-Reidah, Ibrahim M.Liu, ZhaominAdam, TomášLiu, ZhongweiAdams, Sean H.Llabrés, MercèAdamski, JerzyLlorens, EugenioAdela Iuga, CristinaLocatelli, MarcelloAgapiou, AgapiosLogarinho, ElsaAggrey, Samuel E.Lojkova, LeaAksenov, AlexanderLokhov, PetrAlAkwaa, FadhlLomonaco, TommasoAlbano, FrancescoLongnecker, KristaAlbreht, AlenLongo, ValentinaAlessio, NicolaLoo, Ruey LengAlexa-Stratulat, TeodoraLopez Maury, LuisAli, RameezLópez, Lucía MéndezAlieva, Irina BorisovnaLópez, ÓscarAl-Jumeily, DhiyaLopez-Rodas, GerardoAllegra, AlessioLopez-Tello, JorgeAllen, ScottLorenzo, Jose M.Allende, Maria LauraLorkiewicz, Pawel K.Alloatti, GiuseppeLouis, SandrineAl-Mutairi, NawafLu, Chi-YuAlves-Filho, Jose C.Lu, WenyunAlves-Silva, Jorge MLuccini, LuigiAmal, HaithamLuchinat, ClaudioAmato, JussaraLuo, XianAmer, BasharLupette, JosselinAmirabdollahian, FarzadLusczek, Elizabeth R.An, RanMa, ShiningAnathy, VikasMa, ShuanggeAndersen, VibekeMaciejczyk, MateuszAndrej, JanezMacKinnon, NeilAndreoli, RobertaMaglio, CristinaAndreotti, GiuseppinaMahieu, Nathaniel G.Anesi, AndreaMahmood, KhalidAnjos, OféliaMahmud, IqbalAnsai, ToshihiroMairinger, TeresaAntonio, CarlaMaitre, LeaAon, Miguel A.Majumder, Erica L.Arapitsas, PanagiotisMak, TytusArgento, SergioMakino, ToshiakiArias Del Castillo, NataliaMalats, NuriaArias-Santiago, SalvadorMales, ZeljanArita, MasanoriMallouchos, AthanasiosArtati, AnnaMaloney, KatherineArtyukhin, AlexMammi, StefanoAsnicar, FrancescoMandal, Mihir KumarAsquith, Christopher R. M.Mandatori, DomitillaAtilla-Gokcumen, G. EkinManfredi, Kirk P.Aw, WanpingMangalagiu, IonelAzad, Obyedul KalamManier, Sascha K.Azevedo, JorgeMankovsky, BorisBache, SørenMansell, PeterBąchor, RemigiuszMarek, KieliszekBaidoo, EdwardMarginǎ, DenisaBairaktari, EleniMarhuenda-Egea, Frutos CarlosBairaktari, Eleni T.Mariscal, VicenteBais, PreetiMarkić, JoškoBaldanzi, GianlucaMarkley, JohnBalduini, WalterMarkov, GabrielBalgoma, DavidMarkuszewski, MichelBaloni, PriyankaMarnetto, FabianaBaltazar, FátimaMarová, IvanaBandmann, OliverMars, Ruben A.Banerjee, AparajitaMartano, GiuseppeBanfalvi, ZsofiaMartens, JonathanBanoub, Joe H.Martin, EricBarberini, LuigiMartin, MargaritaBarceló-Coblijn, GwendolynMartinez Calejman, CamilaBarding, Gregory A.Martín-Montalvo, AlejandroBartell, Jennifer A.Martín-Peláez, SandraBartelt, AlexanderMatarese, GiuseppeBaruzzi, FedericoMathe, EwyBasetti, MadhuMatsubara, EriBasit, AbdulMatsuda, FumioBasler, GeorgMatulis, DaumantasBattaglia, YuriMaturana, Evangelina López DeBax, Bridget E.Matute-Llorente, ÁngelBeale, DavidMauceri, AngelaBeauchamp, JonathanMazivila, SarmentoBeck, AshleyMcConville, MalcolmBecker, RolandMcgregor, NeilBedia, CarmenMcGregor, Neil R.Behrends, VolkerMcKay, Ryan T.Belenky, PeterMedana, ClaudioBell, RichardMehl, FlorenceBello, Federica DalMeiser, JohannesBeloborodova, Natalia V.Melkani, GirishBel'skaya, LyudmilaMentella, Maria ChiaraBenelli, RobertoMera, PaulaBerdoukas, VasiliosMerino, BeatrizBerg, GabrielaMerlotti, DanielaBerger, Ralf G.Messyasz, BeataBerni, RobertoMetayer-Coustard, SoniaBernillon, StéphaneMethling, KarenBertin, SabrinaMeyer, Markus R.Bertolini, MartaMiceli, VitaleBertram, HanneMiękus, NataliaBertram, Hanne ChristineMijić, PeroBertrand, JustineMikaia, AnzorBertuzzi, TerenzioMikhailova, NataliaBetterle, NicoMikros, EmmanuelBetti, MarcoMilic, NatasaBhanji, Rahima A.Miller, ElżbietaBhupathiraju, ShilpaMiller, Stephen P.Biagi, MarcoMillet, ÓscarBian, ZhaoxiangMills, KenBianco, GiulianaMin, Kyung-JinBiasin, ValentinaMinato, Ken-ichiroBigler, LaurentMiotto, PaulaBikov, AndrasMirzoev, Timur M.Billerbeck, SonjaMishra, DushyantBintsis, ThomasMisra, Biswapriya BiswavasBinz, Tina M.Miyamoto, KojiBird, Matthew J.Mizunoya, WataruBiringer, RogerMlejnek, PetrBirner-Grünberger, RuthMlynarz, PiotrBittremieux, WoutMoise, AlexanderBlackburn, GavinMok, Daniel Kam-WahBlakely, William F.Molina, IsabelBlasco, JosefinaMolinie, RolandBłaszczyk, JanuszMomken, ImanBłaszczyk, LidiaMondelli, DonatoBoard, MaryMonokrousos, NikolaosBoccio, Piero DelMontero, OlimpioBocian, SzymonMonti, MarcoBocianowski, JanMonti, Simona MariaBock, ChristianMontserrat-de La Paz, SergioBogner, WolfgangMoon, Ju-YoungBoguszewicz, LukaszMorales, Jose ManuelBonifay, VincentMorimoto, MasanoriBonora, MassimoMoseley, HunterBoomer, Jonathan S.Moser, Ann B.Botrè, FrancescoMoser, SimoneBoudreau, Paul D.Moškon, MihaBourgoin, SylvainMottis, AdrienneBovet, CédricMouille, GregoryBravo Santos, RafaelMpofu, EliasBrebu, MihaiMuhle-Goll, ClaudiaBrecht, JeffreyMüller, GünterBringel, FrançoiseMunekata, Paulo E.S.Britz-McKibbin, PhilipMuñoz-González, CarolinaBrock, GuyMurata, SoichiroBrodlie, MalcolmMuszyński, SiemowitBroeckling, CoreyMuth-Pawlak, DorothaBroer, StefanMuzykantov, Vladimir R.Bromage, TimothyNaiki-Ito, AyaBroskey, Nicholas T.Najdekr, LukášBrown, Robert J.Nasi, MilenaBrunelli, LauraNassar, Ala. F.Bruzzese, DarioNaviaux, RobertBub, AchimNebija, DashnorBueno Dalto, DanyelNegro, CarmineBui, Duc-CuongNehela, YasserBülow, Margret H.Neufeld, Edward B.Burger, Michael C.Ni, ZhixuBurgess, KarlNielsen, BirtheBussmann, Rainer W.Nielsen, Frederik MølgaardBusta, LucasNiemeijer, Victor M.Busto, RebecaNiizuma, KuniyasuBuszewska-Forajta, MagdalenaNiklison Chirou, Maria VictoriaButts-Wilmsmeyer, Carolyn J.Nikolaev, EugeneCacciola, FrancescoNikolic, DraganaCai, LuNoecker, CeciliaCalado, Cecília R.C.Noga, MarekCamara, Jose SousaNoorani, ImranCampanella, AlessandroNorman, TrevorCampanella, BeatriceNothias, Louis-FélixCampos-Vargas, ReinaldoNovak Kujundžić, RenataCancemi, PatriziaNunemaker, CraigCanela, NúriaNunez, OscarCannazza, GiuseppeOborník, MiroslavCapozzi, VittorioObuobi, Sybil Akua OkyerewaCappellini, FrancescaOda, YoshiyaCappello, TizianaOelkers, PeterCara, Francesca DiOh, HanBinCaraballo-Rodríguez, Andrés MauricioOhtsuka, AkiraCarlos, Ana RitaOliveira, Michelli FariaCarlson, RossOllendorff, VincentCarqueijeiro, InêsOllero, MarioCarradori, SimoneOlson, DanielCaruso, GianlucaOrf, IsabelCasado, NataliaOrso, EvelynCasapullo, AgostinoOrtlund, Eric A.Caspar-Bauguil, SylvieOrtmayr, KarinCastillo, Rafael FernándezOwens, ChristopherCatani, MartinaPacia, Marta Z.Cauli, OmarPadilla-Gonzalez, Guillermo F.Causse, MathildePage, RachelCavaleiro Rufo, JoãoPaglia, GiuseppeCelentano, AntonioPajeva, IlzaChace, Donald H.Pal Choudhuri, ShreoshiChaleckis, RomanasPalermo, AmeliaChamberlain, Casey A.Palička, VladimirChan, Koon HoPalma, MarianaChan, Siu ChiuPandey, Amit V.Chan, StanleyPandey, AparamitaChandler, Joshua D.Pannkuk, EvanChapwanya, AspinasPantea Stoian, AncaChattopadhyay, SaurabhPaolicelli, Rosa C.Checa, AntonioPapadimitriou, Dimitrios T.Cheema, AmritaPapaioannou, TheodoreChekmeneva, ElenaPapassotiriou, Ioannis G.Chen, ChiPapet, IsabelleChen, Yih-YuanParikh, HemangCheng, Chia-WeiParis, DeboraCheng, Hong ShengParry, Traci L.Chicco, AdamPascali, Sandra A. DeChieppa, MarcelloPascottini, Osvaldo BogadoChilds, Gwendolyn VaughnPasolli, EdoardoChin, Young-WonPasquale, CapaccioChiu, Norman H. L.Pastore, IdaCho, Joo-YounPaul, AntoniCho, SayeonPavlova, NatalyaCho, SungeunPawar, SnehalataChoi, Kwon-YoungPedraza Chaverri, JoséChoi, Young HaePeffers, MandyChorfi, YounesPembroke, J.TonyChou, WeichunPentimalli, FrancescaChristensen, ShawnPenzo, MariannaChruszcz, MaksymilianPeptu, CristianChu, Su H.Perdomo, GermanChwil, MirosławaPereira, LeonelCiancio, AurelioPeres, SabineCiani, FrancescaPerestrelo, Rosa Maria De SáCiborowski, MichalPerez De La Vega, MarcelinoCiereszko, IwonaPerez De Souza, LeonardoCimmino, GiovanniPerez-Stable, CarlosCladis, DennisPeris, ManuelClaudio, TrapellaPerkins, AndrewCohen, Arieh SierraPerkovic, Matea NikolacCoker, Robert H.Perl, AndrasColas, CyrilPerła-Kaján, JoannaConsonni, RobertoPeron, GregorioCornett, ClausPerreau, FrancoisCorpas, JavierPescini, DarioCorreia, MarceloPessler, FrankCosta, Rafael S.Peter, KaplanCostantini, LaraPeters, KristianCostantini, SusanPeterson, GregoryCostanzo, MichelePetkova, NadezhdaCostinel, DianaPetriello, Michael C.Cottrell, JeremyPetritis, KonstantinosCottret, LudovicPhan Nguyen, Thuy AnCoughlin, Curtis R.Phan, Chin-SoonCox, JamesPhapale, Prasad B.Cox, JanePhelan, VanessaCozar, IrenePhillips, Gregory J.Cracan, ValentinPhuoc Long, NguyenCrawford, PeterPiasecka, AnnaCreydt, MarinaPickens, Charles AustinCrispo, FabianaPiconese, SilviaCristoni, SimonePierri, Ciro LeonardoCubillos, FranciscoPietruszka, ArkadiuszCuperlovic-Culf, MiroslavaPikula, KonstantinCutignano, AdelePineda, CarmenCutler, RoyPinto, Diana CláudiaCuypers, BartPinto, JoanaCzernek, JiříPinu, Farhana R.Czerwińska, MonikaPisani, FrancescoCzogalla, AleksanderPiska, KamilD’arcy, PádraigPlecita, LydieDaly, RónánPohlentz, GottfriedDamont, AnnelaurePojo, MartaDang, Thu-ThuyPokrywka, AndrzejDaniel, Steven L.Polakof, SergioDaniele, AuroraPolito, AngelaDarcy, JustinPoniedzialek, BarbaraDarras, AnastasiosPoole, DavidDas, AbhirupPospieszna, BarbaraDas, NupurPotaczek, Daniel PDatta, Anita N.Potaczek, Daniel P.Daubon, ThomasPotin, Philippede Aguiar Pertinhez, ThelmaPoulin, RemingtonDe Araujo, Heloisa Sobreiro SelistrePoupot, MaryDe Baere, SiegridPourcher, ThierryDe Bernonville, Thomas DugéPowers, Joseph R.De Graaf, Albert A.Powers, RobertDe Haro, Luis AlejandroPrank, MarjeDe Jong, KirstiePraplan, Arnaud P.De Molon, Rafael ScafPrathipati, Pavan KumarDe Nonneville, AlexandrePrieto, MiguelDe Pinho, Paula GuedesPrieto-Lloret, JesusDe Sanctis, Juan BautistaProbert, FayDe Simone, SalvatoreProchownik, Edward V.De Souza, David P.Prudent, MichelDe Tullio, PascalPugliese, MichelaDeda, OlgaPutri, Sastia PramaDeja, StanislawQazi, SohailDel Ben, MariaQiu, WeiDelaglio, FrankQiu, YunpingDello Sbarba, PersioQuek, Lake-EeDelvin, EdgardQuilichini, TeagenDesagher, SolangeQuilici, DavidDesaire, HeatherQuintás, GuillermoDesguin, BenoîtRadzikowska, UrszulaDi Filippo, MarziaRaftery, DanielDi Gilio, AlessiaRagavan, MukundanDi Lorenzo, FlavianaRago, DanielaDiane Valeriano, ValerieRamadori, GiulianoDias, FranciscaRamalingam, NaveenDiBattista, AliciaRamamoorthy, RengasamyDickens, AlexRamayo-Caldas, YuliaxisDifonzo, GrazianaRamos, PawełDinis-Oliveira, Ricardo JorgeRangel-Huerta, Oscar DanielDittmer, KerenRatajewski, MarcinDjoumbou, YannickRatcliffe, Richard GeorgeDoligalska, MariaRathore, Atul S.Dolinsky, Vernon W.Ratiu, AndreeaDomingo-Almenara, XavierRattray, ZahraDomingo-Fernández, DanielRauckhorst, AdamDonath, Marc Y.Raudenská, MartinaDong, ChuanfuRavandi, AmirDonno, DarioRawel, HarshadraiDos Santos, José Cleiton SousaReis, CristianoDowning, JeffreyRenault, SylvieDrake, RichardReverter, DavidDreux, MarleneReyes-Garcés, NathalyDruzhinina, Irina S.Reynier, PascalDryahina, KseniyaReynolds, ClareDudzik, DanutaRibarova, FannyDufresne, MartinRichling, ElkeDührkop, KaiRichter, SusanDumitru, Croitoru MirceaRiffelmacher, ThomasEbert, BirgittaRigano, FrancescaEcelbarger, Carolyn A.Rioux, VincentEdeas, MarvinRittschof, DanielEghbalnia, Hamid RRizk, Elias B.Eisenreich, WolfgangRižner, Tea LanišnikEitaro, KodaniRizzo, CristianoEl-Bahr, Sabry MohamedRizzo, ManfrediEleutherio, Elis Cristina AraújoRobert, QuinnEliseev, RomanRoberts, MaryElliott, BradleyRobertson, AvrilElmassry, Moamen M.Robinson, Samuel D.Emami, PayamRocchetti, GabrieleEmwas, Abdul-HamidRocha, Hermano A. L.Engel, KathrinRocha, MiguelEnnequin, GaelRodrigues, JoanaEsumi, TomoyaRodrigues, MárcioEszterbauer, EditRodrigues, Pedro MiguelEvans, CharlesRodriguez, JoseEvelyn, RamplerRodríguez-Morató, JoseEverett, JeremyRogers, SimonEykyn, ThomasRogobete, Alexandru FlorinFabien, ForestRolle-Kampczyk, UlrikeFakas, StylianosRomano, PatriziaFalta, DanielRoscini, LucaFan, YongRosebrock, Adam P.Fang, HuangRosenthal, DavidFanos, VassiliosRoss, Richard J.Fariba, FathiRoss, Robert L.Fatima, TahiraRossoni, Luciana VenturiniFattuoni, ClaudiaRouger, CarolineFaure, PhilippeRousu, JuhoFell, DavidRovnyak, DavidFenaille, FrancoisRubakhin, StanislavFerguson, BradleyRubert, JosepFerguson, Jane F.Rubessa, MarcelloFernandes, JolynRudra, PratyaydiptaFernandes, Maria HelenaRuggiero, GiuseppinaFerrari, PietroRuiz, MatthieuFerrieri, Richard A.Ruiz-Rodado, VictorFessler, Michael B.Russell, Steve TFiamoncini, JarleiRusso, NinoFigueiredo, AndreiaRuta, LaviniaFilipiak-Florkiewicz, AgnieszkaRutledge, Douglas N.Finck, Brian N.Ryu, JaihyunkFischer, MarkusSaccenti, EdoardoFleites, Laura A.Sadok, IlonaFlight, Robert M.Sadykov, Marat R.Fokkema, M. Rebecca HeinerSafe, Stephen H.Fonteh, AlfredSaha, RajibForsberg, EricaSaigusa, DaisukeFrandes, MirelaSaini, ShikhaFranke, KatrinSaisho, YoshifumiFraser, DavidSaito, KosukeFreidin, MaximSakamoto, AtsushiFrey, MaximilianSakasai-Sakai, AkikoFröhlich, EleonoreSalek, RezaFrolov, AndrejSalomone, AlbertoFu, XinyuSalto-Gonzalez, RafaelFuchs, BeateŠamec, DunjaFukuba, TatsuhiroSampaio, AnaFukushima, AtsushiSamuelson, JohnFunaki, MakotoSanahuja, IgnasiFunk, Ryan S.Sanak, MarekFuoco, RogerSánchez López, ElenaFurse, SamuelSánchez-Bel, PalomaFürtauer, LisaSánchez-López, ElenaFusco, RobertaSandín-España, PilarGabler, ChristophSansone, AnnaGadea, AliceSantos Júnior, Aníbal De FreitasGagneul, DavidSantos, RenataGai, ZhiboSanz-Paris, AlejandroGalan, JacobSaqui-Salces, MilenaGalaverna, GianniSarasamma, SreejaGallart Ayala, HectorSarma, SauravGallo, Francesca RomanaSayed, Ahmed M.Gamberi, ChiaraSayin, VolkanGambin, AnnaScafidi, JosephGao, BeiScalabrin, ElisaGarattini, EnricoScarpa, MelaniaGarcía Fernández, AntoniaSchlaepfer, Isabel R.García, GabrielaSchnackenberg, LauraGarcia, SergioSchubotz, FlorenceGarcia-Aloy, MarSchüller, ChristophGarcía-Barrado, María JoséSchulz, MargotGarcía-Galbis, Manuel ReigSchulzová, VěraGarcía-Jaramillo, ManuelSchwaiger-Haber, MichaelaGarcía-Pérez, PascualSchwudke, DominikGarcia-Rubio, RocioSciubba, FabioGarikipati, DilipScognamiglio, MonicaGarrett, TimothyScott, AaronGasier, Heath G.Scott-Boyer, Marie-PierGasparri, RobertoSeidl, ClaudiaGastaldelli, AmaliaSengupta, ArjunGauglitz, Julia M.Seo, Dong-OhGaul, DavidSerafini, GianlucaGawlik, AnetaSerefko, AnnaGeist, JuergenSergushichev, AlekseyGençer, NahitSerpa, JacintaGenta-Jouve, GrégorySerrador Peiró, Juan M.Gentili, ValentinaSessa, FrancescoGeorg, RaphaelaSeto, YoshiyaGeorge, AndersonShanbhag, VinitGeorgiadi, AnastasiaShang, ZhuoGevi, FedericaSharma, Bhesh RajGhesquiere, BartSharma, GauravGhosh, ChiranjitShaul, Yoav D.Ghosh, RobinSheikh, BilalGhosh, SoumitaShelake, Rahul MahadevGika, HelenShen, Wen-JunGilardi, FedericaSherrod, StaceyGiles, CoreyShibata, HiroshiGil-Solsona, RubénShimizu, KazuyukiGinebra, María PauShindou, HideoGiordano, ElenaShinozaki, YukikoGirard, ChristianeShirai, YoshinoriGiraudeau, PatrickShizu, RyotaGiron-Gonzalez, Maria D.Shrestha, PushkarGirousse, AmandineShukla, KirtikarGlazier, Douglas S.Siatka, TomášGlobisch, DanielSibley, L. DavidGodos, JustynaSickman, AlbertGodzien, JoannaSidiropoulos, ChristosGoldoni, LucaSilva, Amelia M.Golebiowski, TomaszSilva, PedroGolubnitschaja, OlgaŠimek, PetrGomes, NelsonSimonelig, MartineGómez Castellà, CristinaSimón-Manso, YamilGonçalves, Carlos AlbertoSimonsen, Henrik ToftGoncalves, Luís GafeiraSingh, Brijesh KumarGonzález-Domínguez, RaúlSiniscalco, DarioGonzález-Ruiz, VíctorSinkkonen, JariGöpel, SvenSipos, LászlóGornas, PawelSiristatidis, CharalamposGorodetsky, RaphaelSirvent, RamonGorynski, KrzysztofSisti, DavideGoudarzi, MaryamSitcheran, RaquelGougeon, Régis D.Smith, Charles OwenGouveia, Alexandra M.Smyczyńska, JoannaGovus, AndrewSnelson, MatthewGraça, GonçaloSnowden, StuartGraham, Nicholas A.Sobiech, PrzemysławGraham, StewartSocas, BarbaraGramsbergen, JanSogin, Emilia M.Grant, DavidSong, Hyun-SeobGreatrex, BenSong, MingGreenlief, C. MichaelSoufan, OthmanGriffith, CoreySousa Silva, MartaGrinde, Maria T.South, Paul F.Grison, StéphaneSowa, IreneuszGronwald, WoframSpadafora, Natasha DamianaGrošelj, UrhSpégel, PeterGroten, KarinStanimirova, IvanaGrove, JaneStanislawska-Sachadyn, AnnaGrumet, RebeccaStanković, IvanaGrzegorczyk, IzabelaStankovic, MilanGu, LipingStarčević, AntonioGuan, Xue LiStarowicz, MałgorzataGubert, CarolinaStarz-Gaiano, MichelleGubin, DenisStasiak-Różańska, LidiaGuillevin, RémyStavrianidi, AndreyGunter, Jennifer H.Stefano, AlessandroGünther, UlrichStefanon, BrunoGurgul-Convey, EwaStefanska, AnnaHackl, ThomasStefanuto, Pierre-HuguesHageman, JosSteibliene, VestaHagland, Hanne R.Stepensky, DavidHahn, JuergenStocchero, MatteoHaid, MarkStock, Naomi L.Halama, AnnaStoddard, FrederickHampel, DanielaStorchi, PaoloHan, HongweiStrano-Rossi, SabinaHan, YanStringer, Kathleen A.Hancock, SarahStruck-Lewicka, WiktoriaHanhineva, KatiStuper-Szablewska, KingaHanna-Rose, WendyStyczynski, MarkHannibal, LucianaSulek, KarolinaHano, ChristopheSulżyc-Bielicka, ViolettaHarms, AmySun, WenfeiHashimoto, YoshitakaSun, XiaofeiHatch, Grant M.Sung, JwakyungHattersley, JohnSunny, Nishanth E.Havas, KristinaSurapaneni, Krishna MohanHayama, TadashiSutter, KathrinHeberger, KarolySwarup, SanjayHeiland, InesSweetman, CrystalHeins, Anna-LenaSwierczynski, JulianHerbst-Kralovetz, MelissaSymonds, Michael E.Herminghaus, AnnaSzabados, EszterHerrera-Herrera, AntonioSzopa, AgnieszkaHerrero, EnriqueSzumny, AntoniHeux, StéphanieTait-Woijno, EliaHigashi, RichardTakahashi, MasakiHigham, AndrewTakaya, JunjiHijaz, FarajTakemoto, JonHirschfeld, MarcTaraboletti, AlexandraHitachi, KeisukeTataranno, Maria LuisaHoene, MiriamTaubert, StephenHoffman, JessicaTawfike, AhmedHoffmann, Markus M.Tegeder, IrmgardHoffmann, NilsTelle-Hansen, Vibeke H.Hofmann, Susanna M.Tennstedt, PierreHolcapek, MichalTenori, LeonardoHomolak, JanTenta, RoxaneHong, YanjunTerracciano, RosaHonoré, StéphaneTeschke, RolfHornemann, ThorstenThorsby, Per MedbøeHornung, BastianThorwald, MaxHosokawa, NobukoTian, YuanHossain, EkramTikhonova, IrinaHotea, IonelaTimoshenko, Alexander V.House, ImranTimpani, CaraHowsmon, DanielTimperio, Anna MariaHu, TuoTokarz, JaninaHuan, TaoTokmina-Lukaszewska, MonikaHuang, Chung-GueiTolstikov, VladimirHuang, Shih-YiTolwinski, NicholasHuang, ZhuangrongTomasik, BartłomiejHughes, Chambers C.Tomkin, GeraldHuh, Joo YoungTomkin, Gerald H.Humpel, ChristianTomonaga, ShozoHung, Hsin-YiTong, ShengqiangHunter, Wayne BrianTonutti, PietroHuszcza, EwaToriyama, MichinoriHutley, LindsayTormo, RubenHuuskonen, Mikko TuomasTorné, RamonHuynh, KevinTorrico, DamirHwang, In KooTotta, PierangelaHwu, Wuh-LiangToubiana, DavidHyötyläinen, TuuliaToya, YoshihiroIcyuz, MertToyoshima, MasakazuIdborg, HelenaTrainor, PatrickIelciu, IrinaTrentini, AlessandroIglesias-Osma, Maria CarmenTres, Marcus ViniciusIjiri, DaichiTrico, DomenicoIkonomopoulou, MariaTrifonova, Oxana P.Inchingolo, FrancescoTrimigno, AlessiaInfantino, VittoriaTrognitz, FriederikeIngram-Smith, CherylTroisi, JacopoIonete, Roxana ElenaTroncoso-Ponce, AdrianIshii, IsaoTsakovska, IvankaIslam, Mohammad MazharulTsaniklidis, GeorgiosIuga, DinuTsentalovich, Yuri P.Ivanov, AlexanderTsiamis, GeorgeIvanov, BorisTsigelny, Igor F.Iwamoto, NaokiTu, ZhidongIzquierdo-García, José LuisTufarelli, VincenzoIzumi, YoshihiroTurano, PaolaJaburek, MartinTuri, Kedir NeshaJacob, ChristopheTürkoğlu, OnurJacobi, SheliaTuvdendorj, DemidmaaJaffe, EileenTuzimski, TomaszJahandideh, ForoughTyszka-Czochara, MalgorzataJang, CholsoonUeda, ShujiJans, Judith J.M.Ueno, YujiJaumot, JoaquimUhle, FlorianJawad, Ali S.M. Ulaszewska-Tarantino, MarynkaJayaraman, ArulUlmer, Candice Z.Jenkins, BenjaminUmano, Giuseppina RosariaJerin, AlešUmehara, MikihisaJerkowić, IgorUnderwood, Mark D.Jeucken, AikeUngurianu, AncaJiang, ChunjieUtsumi, YoshinoriJiang, Shu-YeValdés López, OswaldoJiang, XiqianVališ, KarelJiang, XuntianVan Den Bossche, JanJin, GeVan Gastel, NickJiri, VrbaVan Meulebroek, LievenJohansson, BjörnVan Raemdonck, KatrienJohn A. RathmacherVan Ravenzwaay, BennardJohnson, James D.Van Rijn, Bas B.Jolicoeur, MarioVanhaecke, LynnJones, J. AndrewVaniya, ArpanaJones, Oliver A. H.Vaquero, María PilarJones, PatriceVaradarajan, ShankarJørgensen, KirstenVaranoske, AlyssaJose, Silva-NunesVargas Jentzsch, PaulJoske, David J.L.Vasin, Mikhail V.Joven, JorgeVazquez, AlexeiJullian, NathalieVázquez-Baeza, YoshikiJulve, JosepVelena, AstridaJunot, ChristopheVemula, HarikaKaina, BerndVenkat R, PannalaKaiser, PeterVeres, SzilviaKallscheuer, NicolaiVergara, FreddKammerer, BerndVermathen, MartinaKanak, Mazhar A.Vermathen, PeterKaneko, GenVersino, ElisabettaKang, Kyo BinVerstraete, AlainKang, Min-JungVervoort, JacquesKaňková, KateřinaVignoli, AlessiaKaragiannis, EvangelosVillafuerte-Gálvez, Javier A.Karagouni, Amalia D.Villas-Boas, Silas G.Kar-Chun, TanVillaseñor, AlmaKärkkäinen, OlliVincenti, MarcoKårlund, AnnaVitrac, HeidiKarpe, Avinash V.Voy, BrynnKashfi, KhosrowVukicevic, SlobodanKasimanickam, RamanathanWadsack, ChristianKasture, AmeyaWahl, Daniel R.Kaszowska, MartaWahli, WalterKaufmann, MartinWakayama, MasatakaKaushik, AkashWaldherr, SteffenKawano, NatsukoWalf, Alicia A.Ke, Liang-YinWalker, Douglas G.Kebede, MelkamWalker, HeatherKechris, Katerina J.Wallace, M. ArielKeinänen, MarkkuWanders, Ronald J.A.Keisaku, SatoWandy, JoeKelly, RachelWang, GuanKendall, AlexandraWang, NanKenéz, ÁkosWang, PingyuanKeser, TomaWang, San-YuanKhadka, ManojWang, XiaobinKhajeh, EliasWang, XushengKhan, MerajuddinWang, YingKhozin-Goldberg, InnaWang-Sattler, RuiKhroustalyova, GalinaWant, Muzamil YaqubKieliszek, MarekWard, Kristen M.Kikionis, StefanosWatanabe, MikikoKikuchi, HidehikoWatson, DavidKilk, KalleWeaver, ConnieKim, Jae KwangWeichhart, ThomasKim, JaeheeWeinhold, AlexanderKim, SeonghoWelch, KennethKim, Won HoWeljie, AalimKim, Young-zoonWendell, Stacy GelhausKindt, AlidaWesterhuis, Johan A.Kirwan, JenniferWeston, Leslie A.Klein, JochenWeston, PaulKlupczynska, AgnieszkaWezgowiec, JoannaKnight, JohnWhite, PhillipKnittelfelder, OskarWhitelegge, JulianKobayashi, MasakiWhitfield, Phil.Koch, BenjaminWijeyesekera, AnishaKoelmel, JeremyWijk, Rob Christiaan VanKöfeler, Harald C.Wilde, Michael J.Kokotou, Maroula G.Willers, ClarissaKolmert, JohanWilliams, Robert J.Kopeć, AnetaWilliams, TomKopec, RachelWillinger, TimKornberg, Michael D.Wilson, Philippe B.Kos, KatarinaWińska, KatarzynaKoshino, HiroyukiWinter, GalKosyakov, Dmitry S.Winum, Jean-YvesKotanidou, AnastasiaWojakowska, AnnaKotwal, AnupamWojciuk, BartoszKoudounas, KonstantinosWojnowski, WojciechKoulman, AlbertWojtunik-Kulesza, KarolinaKovacs, RenatoWojtunik-Kulesza, Karolina A.Kozłowska, MariolaWoltering, ErnstKremer, WernerWong, AlanKremling, AndreasWood, Paul L.Křížová, LudmilaWood, Troy D.Krzyżanowska-Kowalczyk, JustynaWoods, Jon P.Ku, Kang-MoWoodward, SteveKuda, OndrejWooton-Kee, Clavia RuthKudłak, BłażejWormser, UriKules, JosipaWouter H. Lamers Kuligowski, JuliaWu, ChaoKullaa, ArjaWu, SiKultima, KimWu, SonglinKumakura, NaoyoshiXia, JianguoKumar, VikasXiao, WangKupcewicz, BogumiłaXiao, ZhengtaoKurka, OndrejXing, ZhuoKuryłowicz, AlinaXu, KuiKwon, Sung WonYadav, DhananjayLa Barbera, GiorgiaYahiroda, HidekiLa Frano, MichaelYamaguchi, YusukeLacal, Juan CarlosYanes, OscarLaghi, LucaYang, Kuang-YaoLai, Zon WengYang, Seung HwanLaiakis, EvageliaYang, Seung-HoLamichhane, SantoshYang, Shi YingLamy, ElsaYang, Wei-hsiungLancaster, Graeme I.Yanshole, Lyudmila V.Lara, Isabel Yanshole, VadimLarrosa, MarYao, Pei-LiLasky-Su, JessicaYasuda, KazukiLau, ChunghoYazbeck, RogerLauberts, MarisYik, JasperLaurent, MichaëlYilmaz, AliLe Gall, GwenaelleYin, ZhiminLe Roy, CarolineYoo, Hyun JuLebaron, SimonYoshigaki, JunkoLee, Choong HwanYu, JiujiuLee, Do YupYu, JornLee, Dong-YupYu, NingLee, EunkyungYu, Yu-HsiangLee, KyuwanYuan, RongLee, SunjaeYuzbashev, TigranLee, Tzu-TaiZabiegała, BożenaLee, Wing-KeeZadra, ClaudiaLeenders, JustineZager, JordanLehotský, JánZampieri, MattiaLei, ZhentianZan, HongLeigh, Brittany A.Zandberg, WesleyLemaitre, Rozenn N.Zaragoza, WilliamLemos, Bruno SilvaZarrouk, OlfaLeon, CarlosZawadowicz, Maria A.Leoni, ValerioZellers, Edward T.Levesque, EricZernii, Evgeni YuLi, DongyingZhang, BoLi, Fu-shuangZhang, FangLi, GuannanZhang, GuannanLi, GuohuiZhang, KaiLi, LingZhang, YoujunLi, MingjiZhao, LiangLi, ShuzhaoZheng, JiaminLi, SunanZheng, XiaorongLiagre, BertrandZhu, JiangjiangLiatis, StavrosZhu, LinLicata, MarioZhu, XueweiLiddelow, ShaneZielinski, Daniel C.Liebal, UlfZierau, OliverLiechti, George W.Zille, MariettaLiggi, SoniaZińczuk, JustynaLim, HyungyuZiouzenkova, OulianaLin, Cheng-HuangŻołądek, TeresaLin, FanZorzan, SimoneLin, Ying-ChiZucker, JeremyLindqvist, HelenZurth, ChristianLionetto, Maria GiuliaZuther, EllenLiotti, Antonietta


